# Sexual and reproductive healthcare utilisation and affordability for South Sudanese refugees and host populations in Northern Uganda: A mixed methods study

**DOI:** 10.1371/journal.pgph.0002351

**Published:** 2023-09-06

**Authors:** Pallavi Prabhakar, Neha S. Singh, Munshi Sulaiman, Jessica King, Zia Saddique, Sandra Mounier-Jack, Barbara Asinde, Sylvia Namakula, Josephine Namatovu, Rogers Kapiti, Joram Kasiri, Josephine Borghi

**Affiliations:** 1 Independent Evaluation and Research Cell, BRAC Uganda, Kampala, Uganda; 2 Department of Global Health and Development, Faculty of Public Health and Policy, London School of Hygiene and Tropical Medicine, London, United Kingdom; 3 Department of Health Services Research and Policy, Faculty of Public Health and Policy, London School of Hygiene and Tropical Medicine, London, United Kingdom; 4 Independent Consultant, Kampala, Uganda; Asian University for Women, BANGLADESH

## Abstract

Given Uganda’s increasing refugee population, the health financing burden on refugee and host populations is likely to increase because Uganda’s integrated health system caters to both populations. We used sexual, reproductive, and maternal health (SRMH) as a lens to assess the utilisation and user cost of health services in Northern Uganda to identify potential gaps in SRMH services and their financing. We conducted a cross-sectional survey among 2,533 refugee and host women and girls in Arua and Kiryandongo districts. We conducted 35 focus group discussions and 131 in-depth interviews with host and South Sudanese refugees, community members, health workers, NGO and governmental actors. Qualitative data were analysed thematically using a framework approach. Quantitative data were analysed using t-test, chi-square tests, multivariate logistical regression, and a two-part model. We found high levels of access to maternal care services among refugee and host communities in Northern Uganda, but lower levels of met need for family planning (FP). Refugees had higher uptake of delivery care than host communities due to better-resourced refugee facilities, but incurred higher costs for delivery kits and food and less for transport due to facilities being closer. FP uptake was low for both groups due to perceived risks, cultural and religious beliefs, and lack of agency for most women. Host communities lack access to essential maternal healthcare services relative to refugees, especially for delivery care. Greater investment is needed to increase the number of host facilities, improve the quality of SRMH services provided, and further enhance delivery care access among host communities. Ongoing funding of delivery kits across all communities is needed and new financing mechanisms should be developed to support non-medical costs for deliveries, which our study found to be substantial in our study. All populations must be engaged in co-designing improved strategies to meet their FP needs.

## Introduction

Conflict, violence, and climate change forcibly displaced 89.3 million people globally by the end of 2021, of which 27 million were refugees [1]. 85% of these refugees were hosted in developing countries, creating pressures on host countries’ health systems which ultimately constrained access to essential healthcare services by both refugee and host populations. Ensuring effective access to care for refugees while ensuring that host populations access is not compromised will be key to achieving continued progress towards universal health coverage goals. Currently, around half of refugees are women, to whom access to affordable and equitable sexual, reproductive and maternal health care (SRMH) services is particularly important as it serves as a safeguard to unwanted pregnancies, sexually transmitted infections, neonatal and maternal mortality [2]. However, evidence suggests that refugee women and girls often lack access to affordable SRMH.

In a systematic review of studies exploring knowledge, access, and experience of sexual and reproductive health services, Ivanova et al 2018 found that access remained low among refugee young women and girls in Africa due to long distances, costs, and stigma [2]. They also highlight the poor quality of services including stock outs, lack of acceptable and affordable contraceptive services and language barriers. Lack of access to essential SRMH leads to poor service utilisation, further exacerbating concerns to achieving universal health coverage (UHC) in host countries. Two recent systematic reviews also point towards the scarcity of evidence and data on utilisation of key SRMH services in refugee settings [3, 4]. Additionally, most of the existing evidence on service access and utilisation remains limited to the study of refugee women and girls only, missing out on relative comparison with host communities [5, 6]. Although, an emerging literature explored the differences in access to quality antenatal and delivery care [7] and costs [5, 8] between refugees and host communities in Uganda, these studies were based only on quantitative data and did not explore the driver of differences using mixed methods research.

Uganda offers an interesting context for studying access to care for refugees and host communities because of the existence of a unique Comprehensive Refugee Response Framework (CRRF). This framework ensures integrated health service delivery to both refugees and host populations to achieve UHC, and is regarded as one of the most progressive in the world in terms of integrating refugees. Uganda is also the third largest refugee hosting nation globally, and the largest in Africa [9]. As of 16^th^ June 2022, Uganda was hosting 1.5 million refugees, mostly in the West Nile, Northern, and Western parts of the country. However, even after the introduction of CRRF, refugees continue to benefit from donor investments which could improve access or lower healthcare costs relative to host populations. Refugees equally suffer from informational, cultural or language barriers which might hamper access.

The key objective of our study is to describe and compare utilisation and costs of SRMH services for refugees and host women from the same communities in Uganda and explore drivers of eventual differences using a mixed methods approach. We also compare these outcomes across two different settings in Uganda—Arua and Kiryandongo districts, where we have important variation in terms of distance to health facilities, time of arrival of refugees in Uganda, languages used for health service delivery, number of health facilities and geographical context, all of which have effects on utilisation and costs of SRMH care.

## Methods

### Country context

#### Refugee and host population

Refugees in Uganda come from 10 countries, though the majority are from South Sudan and the Democratic Republic of Congo (DRC). As of 20^th^ June 2022, Uganda hosted 927,823 refugees from South Sudan. Although Uganda’s refugee profile was disproportionately female in early years of hosting refugees, this proportion reduced to 52% as displacement became more protracted [10]. Over 61% of Uganda’s refugee population is less than 17 years of age [10], and 25% are under the age of 5 years [11].

A recent World Bank survey representative of refugee and host populations in Uganda found that despite feeling secure and welcome, refugees live in precarious conditions [11]. Findings from the survey suggested that almost half of the refugee population (48%) in the country are living in poverty, compared to 17% of host populations. Poverty among refugees is highest in the West Nile region where close to 60% of refugees are poor and around 30% of hosts are poor. Food security is a concern for both refugee and host households, with 70% of refugee households experiencing severe food insecurity, compared to 50% of host households. The high dependency ratios of refugee households exacerbate the risks to wellbeing–within refugees, there are about 1.7 dependent members for every non-dependent member, compared to 1.2 for hosts [11].

#### Healthcare system

The healthcare system in Uganda is comprised of both public and private sector. Private sector organisations include Private Not for Profit (PNFPs) (e.g., faith-based Catholic, Protestant and Muslim Medical Bureaus), Private for profit, Health Practitioners (PHPs) and Traditional Contemporary Medicine Practitioners (TCMPs). The public sector includes Government health facilities, National Drug Authority and Medical Stores. Community outreach and first point of contact services are provided by the Village Health Teams (VHTs), which are the public volunteers in each village. The public sector provides primary preventive, promotive and outpatient curative health services at the Health Centre- II and Health Centre-III level. These include regular antenatal care, outreach and immunization services. In addition to these services, Health Centre-IIIs offer maternity, inpatient health services and laboratory services. Emergency surgeries, blood transfusions, consultations and laboratory services are offered at the level of Health Centre- IV and General Hospitals. Referral hospitals provide additional services like psychiatry, ear, nose and throat (ENT), ophthalmology, dentistry, intensive care, radiology, pathology, specialized surgical and medical services [12].

The Government run facilities provide free services with no user fees. This contrasts with the private clinics and pharmacies which charge a user fee. However, populations purchase drugs and commodities from private pharmacies when there are drug stock outs at government-owned health facilities.

Most of the hosts and refugees either walk to the health centres or travel by a boda-boda (motorcycle). In case the ambulance service is unavailable, transport costs for referral are borne by the communities themselves.

#### Healthcare financing

There is a heavy dependency on donors and the private sector for financing healthcare services in Uganda. The share of public spending on the healthcare sector was a mere 5.1% for the financial year 2020–2021 [13]. Only 6% of the already limited health budget was allocated to RMNCH care in 2019. It is reported that during 2019, donor’s contributions amounted to 76% of the total money in the health sector envelope, excluding private contributions [13]. Development partners such as Medical Teams International, Save the Children, ACORD, UNHCR, UNFPA, UNICEF and others also provide support in the form of grants, skilled staff, technical assistance, ambulance service, medical equipment and outreach programmes for the health facilities. A few of the health facilities in host and refugee settlements are also supported through a World Bank funded Results Based Financing (RBF) scheme. Some government-run facilities operate in the form of a partnership with a private ward/wing. For example, Kiryandongo hospital has a private wing having independent charges for the services obtained.

The Ministry of Health, Uganda provides free Mama kits to mothers who deliver at the facility, free of charge. Each Mama kit contains a plastic sheet, sterile gloves, razor blades, cord ligature, cotton, sanitary pads, tetracycline and soap. Dignity kits are provided to displaced mothers by UNFPA, to restore their “dignity” for seeking reproductive healthcare. They typically contain basic hygiene items such as toothbrushes, toothpaste, sanitary napkins, underwear, towels, soap and, depending on the needs and cultural norms of affected populations, buckets, slippers, and headscarves—items that at the time were not normally distributed in humanitarian aid settings. Both kits contain items but are not limited to what women need at the time of their delivery. Even though both kits are meant to be free of charge, stock outs are common, and women end up paying for accessing the kits at the time of delivery. Dignity kits are primarily delivered at the Health Centres inside the Refugee Settlements whereas Mama kits are primarily delivered at the Health Centres inside the Host Villages, but they are available to all populations.

#### Study setting

**Fig 1:**
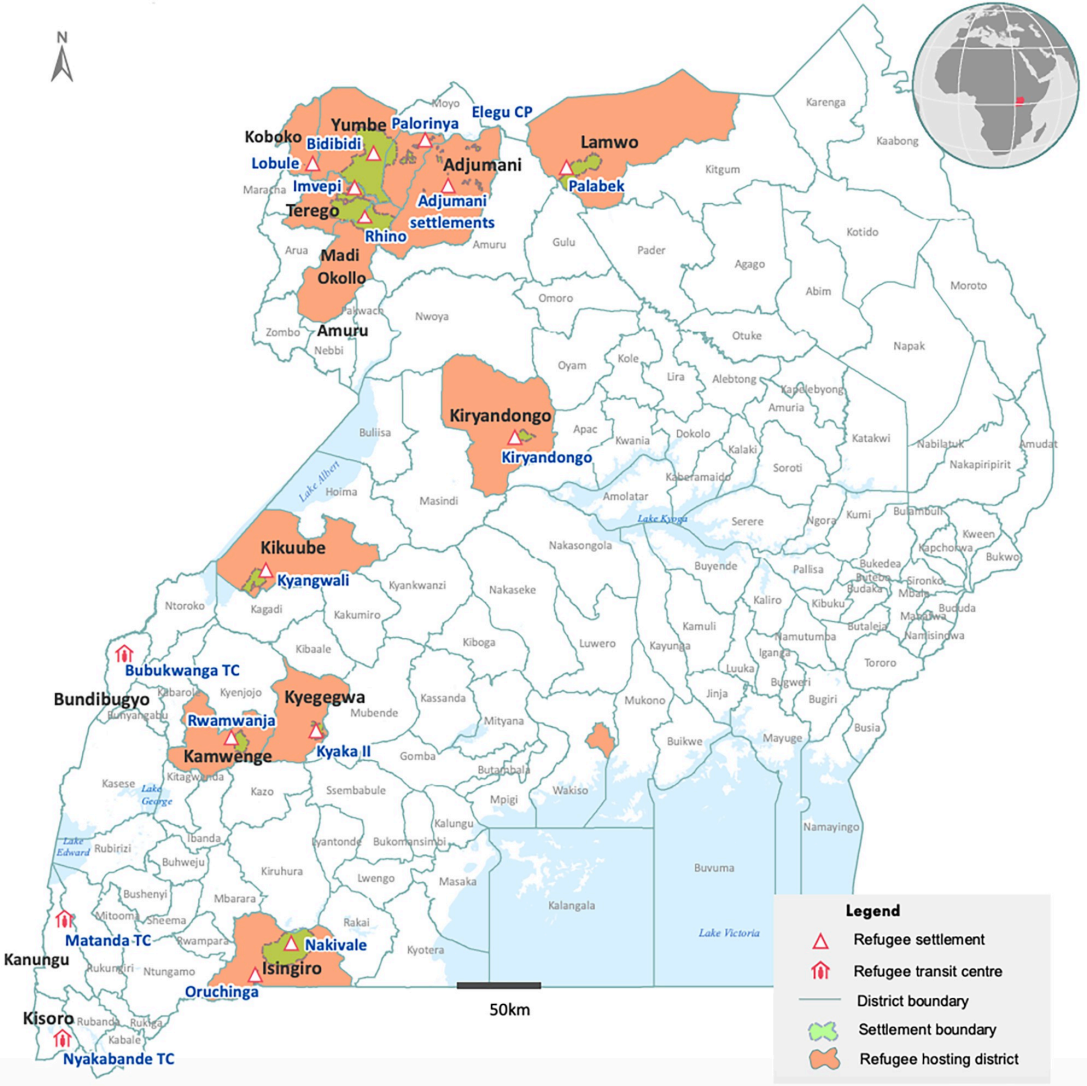
Map of refugee hosting districts in Uganda as of August 2020 [13].

This study was conducted in host and refugee settlements in Arua (current Madi Okollo and Arua district) and Kiryandongo districts in Uganda (Fig 1). Refugees were selected from Kiryandongo refugee settlement in Kiryandongo and Rhino Camp refugee settlement in Arua. The districts are unique to study because they are not only among the top six Ugandan districts hosting a majority of South Sudanese refugees, but also have significant variation between them in terms of (i) time of arrival of refugees, with Arua having more recently resettled refugees compared to Kiryandongo (ii) distance of the camp from the main town as Arua is much further from the town compared to Kiryandongo and (iii) number of health facilities, as Arua has more health facilities compared to Kiryandongo.

During 2016–17, there was a rapid influx of South Sudanese refugees in Uganda. More than 90% of these refugees were settled in the refugee settlements in Yumbe, Adjumani, Moyo (current Obongi) and Arua (current Arua, Madi-Okello and Terego) districts. As of 1^st^ April 2016, Arua was hosting 32,490 refugees compared to 52,534 refugees in Kiryandongo [14]. At the time of the study, (July 2019) the number of refugees had increased by 1.2 times (n = 61,011) in Kiryandongo and by 5 times in Arua (n = 60,550 in Imvepi and n = 110,345 in Rhino Camp) from 2016 levels [15]. Overall, Arua has more number of refugees and a greater number of donor funded health facilities as compared to the Kiryandongo Refugee Settlement.

Additional characteristics of both the districts are outlined in Table 1.

**Table 1:** Characteristics of Arua and Kiryandongo districts.

	Arua district	Kiryandongo district
*Location*		
Region	North-western Uganda	Mid-western Uganda
Land Area	4,274.13 km^2^	3,624.1 km^2^
Distance from Kampala	520km	218km
Refugee settlements	Imvepi, Omugo, Rhino Camp Settlement	Kiryandongo refugee settlement
Distance of the refugee settlement from main town	70km	5km
*Population Composition*		
Number of Sub-counties	18	4
Number of Villages	1044	236
Population- Host (July 2019) [16, 17]	783,769	266,197
Population- Refugee settlement in focus (July 2019)	110,345	61,011
*Health facilities [18]*	102	39
*Of which*:		
Number of General Hospitals	4	2
Number of Regional Referral Hospitals	1	0
Number of Health Centre—II	32	15
Number of Health Centre- III	40	8
Number of Health Centre- IV	3	0
Number of Clinics	22	14

The study targeted refugees and host populations living predominantly in the rural settings. Both communities engage in subsistence farming, livestock rearing, boda-boda (motorbike) driving and small-scale business activities for earning a living. Refugees also benefit from 31,000 UGX (USD 8.3) cash per capita or food package of an equivalent amount from the World Food Programme.

Kiryandongo General Hospital in Bweyale town and the Arua Regional Referral Hospital in Arua town are the two main Government hospitals accessed by refugees in the two districts. Other healthcare providers can be accessed by both refugee and host populations [19].

We defined a host health centre as one that is primarily located in host villages and refugee health centres as those that are in refugee settlements. In some cases, both types of facilities are in or near a refugee settlement and can be accessed by both hosts and refugees. Both types of facilities are government-owned, even if they have been given additional resources by external actors (e.g., non-governmental organisations or multilateral agencies).

### Study design

The study employed a mixed method approach by undertaking data collection via a cross-sectional survey, focus group discussions (FGDs) and semi-structured interviews (SSIs).

#### Quantitative data

The quantitative analysis aimed at examining the level of access to reproductive and maternal care services and where these services are accessed, together with their costs, and the extent to which these outcomes differed between refugee and host populations and other population characteristics.

*Sampling and data collection*. A sample of 2,533 women of reproductive age (15–49 years) across the refugee and host communities in Arua and Kiryandongo was surveyed during July 2019. Refugees in the Rhino Camp Settlement and Kiryandongo Settlement reside in clusters whereas the host communities reside in villages, which are the lowest administrative level in Uganda and typically comprise of 50–70 households [20]. For sampling purposes, a village and a cluster were taken to be the same, and hereafter we refer to villages. The Integrated Refugee Response Framework permits refugees and host communities to live together. A village was categorized as a ‘host’ if more than 50% of the population was of Ugandan nationals; and ‘refugee’ if more than 50% of the population was of refugees. A household was classified as an entity comprising of individuals who live in the same house and who have common arrangements for basic domestic activities such as cooking and eating [21].

Two-staged sampling method was used for sample selection. At the first stage, 20 villages were randomly selected from a list of all the refugee and host villages in Arua and Kiryandongo respectively. The list of refugee clusters was obtained from the Office of the Prime Minister, Uganda in and that of Ugandan Villages was obtained from the District offices of Arua and Kiryandongo. During the second stage, the enumerators followed a random walk [22] to interview a sample of 30 households from each village which had at least one woman of reproductive age (15–49 years). In case more than one woman of reproductive age was present in the household, the KISH grid method was used to identify the respondent randomly [23]. Thus, only one woman from each household was interviewed. The research team only sampled refugees from refugee villages and Ugandan nationals from the host villages.

Sample size calculation to detect the difference in utilisation of key SRMH services between refugee and host groups was done using the formula:

$$n=\frac{1+\rho (m-1)*Z^{2}P(1-P)}{E^{2}}$$

where n = sample size, z = value of the desired confidence interval, p = estimated proportion of an attribute present in the population, *ρ* = intracluster correlation, m = number of individual in each cluster (village), and e = desired level of precision the researcher is willing to accept [24]. Assuming p = 0.5, margin of acceptable error I to be 4%, confidence interval at 95% level (z = 1.96), m = 30 and *ρ* = 0.10 (design effect = 3.9), the required sample size was estimated to be approximately 2400, with 1200 respondents in each district, and 1200 refugees and 1200 hosts across the sample [25].

Over the course of the study, the research team interviewed greater number of respondents to adjust for a potential non-response rate of 8%. The final sample size of the study is presented in Table 2.

**Table 2:** Number of respondents interviewed by district and refugee status.

	Total	Arua	Kiryandongo
Host community	1182 (40 villages)	575 (20 villages)	607 (20 villages)
Refugee settlement	1351 (40 villages)	698 (20 villages)	653 (20 villages)
Total	2533	1273	1260

The questionnaire was administered on Survey CTO [26] in English and Arabic and was 40–45 minutes long. Data were collected by 25 trained enumerators over a period of 30 days from July- August 2019. Informed consent was sought, and the respondents were free to withdraw or refuse their participation at any point of the survey process. Confidentiality was always ensured and data analysis was undertaken with anonymised data and the enumerators ensured privacy while asking about personal details and sensitive topics including the sexual and marital history of the respondents.

*Outcomes*. The survey captured information on health care utilisation of antenatal care, delivery care and family planning services for women of reproductive age. Information was also collected on health care expenditures associated with care seeking.

*Health care utilisation*. Women who gave birth in Uganda during the two years preceding the survey were asked about the number of ANC visits, the timing of the first ANC visit, and where they most frequently sought care during their last pregnancy. Outcomes of interest were receipt of four or more ANC visits and seeking care during the first 12 weeks of the pregnancy (first trimester), and place of care seeking. Respondents were also asked if they delivered their last child at a health facility and the place of delivery. In addition, information was collected on whether they had received a Mama kit or Dignity kit.

Women were questioned on the use of any contraceptive methods for delaying pregnancy. We used the Demographic and Health Survey (DHS) definition for met need for contraception, which is defined as any sexually active (non-pregnant) women using any form of contraceptive methods for spacing or limiting [27]. For women using any form of contraception, information on the type of method used was also collected. These were classified into modern and traditional methods. Modern methods included oral contraceptive pills, hormonal injections, intra-uterine device, Hormonal implants, male and female condoms and ring. Traditional methods constituted periodic abstinence (rhythm), withdrawal, use of traditional herbs or medicines and prolonged breastfeeding.

*Costs of care*. Women who had at least one ANC visit were asked about the components of ANC received at the facility for their last pregnancy (e.g. tetanus injection, HIV screening, blood test, urine sample collection, ultrasound, malaria, medicines and iron tablets) and the fees associated with each. Medical costs of ANC were calculated by obtaining a sum of the costs of each of these components, restricted to those who sought care at a formal health facility. This implies accessing care at the health centres, hospitals and private clinics.

For deliveries occurring at formal facilities, respondents were asked to report on the medical (consultation fees, expenditures on drugs, supplies and delivery kits), non-medical costs (food) of care, and transport costs.

Respondents using contraceptive methods were also asked about their costs and frequency of use.

*Sample characteristics*. We also captured information on the characteristics of the female respondent (age, occupation, education, religion, country of origin) and their household (socio-economic status, household size).

*Data analysis*. Data were exported from Survey CTO [26] to Stata version 15 [28] for analysis. Descriptive analysis of associations between refugee status and the outcomes of interest were conducted using Chi Square test for binary outcomes and t-test for continuous outcomes. Multivariate logistic regression (with standard errors clustered at village level) was used to analyse if the differences in service utilisation by refugee status and districts, sustain upon controlling for participant and their household characteristics.

A two-part model (with clustered standard errors) was used to examine the effect of refugee status and districts on likelihood for payment and costs for those who paid to access antenatal, delivery and contraceptive services upon controlling for participant and their household characteristics. The first part modelled the likelihood of paying for essential services using a multivariate logistic regression with clustered standard errors. The second part modelled costs for those who paid using GLM regression with gamma family and log link function. We report the results of multivariate logistic regressions in the form of marginal effects and used the following general specification for the regression models:

$$Y_{i}=\alpha +\beta R_{i}+\gamma D_{i}+\mu (R*D)+\partial Z_{i}+\varepsilon_{i}$$

where,

Y_i_ = Outcome of interest

R_i_ = 1 for refugee respondents and 0 for host respondents

D_i_ = 1 if district is Arua and 0 if it is Kiryandongo

R*D = Interaction term between District (Arua/Kiryandongo) and Refugee status (Refugee/Host)

Z_i_ = Vector of participant and household characteristics as control

*ε_i_* = Error term

#### Qualitative research

*Recruitment and data collection*. In order to understand the “how” and “why” access and costs might differ between refugee and host communities and to understand how the provision of care to refugees might have knock on effects for hosts, we conducted focus group discussions (FGDs) and semi-structured interviews (SSIs) with local stakeholders (local authorities, NGOs, UN and multilateral agencies, health workers, religious leaders), as well as South Sudanese refugees living inside and outside refugee settlements in Kiryandongo and Arua. We conducted 34 FGDs and 129 SSIs (Table 3).

**Table 3:** Number of focus group discussion and semi-structured interviews by respondent type.

Respondents	Arua		Kiryandongo		Total	
	FGD	SSI	FGD	SSI	FGD	SSI
Local governmental authorities	1	3	2	3	3	6
NGOs, UN and multilateral agencies	0	8	1	4	1	12
Health workers from both refugee and governmental health facilities	3	9	1	9	4	18
Informal health workers (TBAs, volunteer community outreach workers)	0	4	0	3	0	7
Religious leaders–South Sudanese	0	2	0	2	0	4
Religious leaders–Host population	0	2	0	2	0	4
South Sudanese refugees—male	3	13	3	13	6	26
South Sudanese refugees—female	4	14	4	14	8	28
Host population—male	3	6	3	6	6	12
Host population—female	3	6	3	6	6	12
Total					34	129

We identified South Sudanese refugees and host community members via the following two channels: (i) ongoing BRAC programmes within Arua and Kiryandongo and (2) community representatives and local community groups. We identified other local stakeholders using snowball sampling and investigator contacts. To participate, refugees needed to be of South Sudanese origin and registered within Arua and Kiryandongo refugee settlements. Local stakeholders needed to be working in or involved in governance or service provision for refugees in Arua and Kiryandongo refugee settlements. Potential participants were given an information sheet written in the vernacular language for those who were not fluent in English, fully detailing the study objectives and explaining all aspects of participation, including the right to withdraw from the research.

Qualitative research procedures included a member of the research team introducing the respondent to the study, its objectives and the KII or FGD to take place. If they agreed to take part, the study team members obtained written consent from the respondents. Seven study team members (NSS, PP, SMJ, SN, JN, JK, RK) then conducted KIIs or a FGDs following a semi-structured interview guide in English or relevant local languages depending on interviewees’ preferences, using interpreters if needed to facilitate translation. KIIs and FGDs were audio recorded and reflective notes were taken. KIIs and FGDs were conducted in a private location convenient for participants, and in quiet environments away from clinical areas for health workers. KIIs lasted between 45–60 minutes, and FGDs lasted between 50–90 minutes. South Sudanese refugees and host populations were compensated with 29,000 UGX (approximately $8 USD) for their time to participate in the study.

South Sudanese refugees and host populations were asked about their experiences of sexual and reproductive health care access, utilisation, cost, and barriers and facilitators to care seeking; and local authorities and stakeholders were asked about the financing, governance and/or provision of sexual and reproductive health services to refugee and host populations. Interview topic guides were developed for this study after pilot testing with respondent groups.

*Data analysis*. Interviews were transcribed verbatim and analysed thematically in NVivo 13 software [29] using the following stages outlined by Braun and Clarke [30]: data familiarisation, coding and theme identification and refinement. Analytical rigour was enhanced by NSS and SMJ discussing coding approaches and data interpretations. Interviews were coded using a framework approach whereby a priori and emerging themes were applied.

We followed Noble and Smith’s recommended steps to enhance the validity and reliability of qualitative data collection and analysis, including accounting for personal biases, frequent communication amongst the qualitative study team (NSS, PP, SMJ, SN, JN, JK, RK), and ongoing critical reflection of methods to ensure sufficient depth and relevance of data collection and analysis [31].

### Data triangulation and synthesis

We triangulated qualitative and quantitative findings and synthesised results following principles of mixed methods research outlined by Fetters et al [32]. At the data interpretation and reporting stage, we used the weaving approach which involves writing both qualitative and quantitative findings together on a theme-by-theme basis.

### Ethics statement

Ethical approval was obtained from the Institutional Review Boards of Makerere School of Public Health in Uganda (reference number 680) and the London School of Hygiene and Tropical Medicine in the United Kingdom (reference number 16440). The study was registered with Uganda National Council of Science and Technology (UNCST) with the reference number SS296ES. Written informed consent with signatures from literate participants and a thumbprint from illiterate participants along with a witness’ signature was obtained from all study participants, and from the parent/guardian of each participant under 18 years of age.

## Results

### Characteristics of survey participants

The mean age for the host and refugee women in the cross-sectional survey was 25 years (Table 4). However, there were numerous differences between host and refugee households. Female refugees were more likely to have no education, although they were also more likely to have secondary education and above than their host counterparts. They were more likely to come from large households, have a female household head, and were less likely to be working than their host counterparts (Table 4). In terms of socio-economic indicators, refugee households were more likely to have suffered food insecurity and be benefiting from cash or other benefits from development organizations. Explained by their entitlement to receive cash or food benefits from the World Food Programme (WFP).

**Table 4:** Socio-demographic characteristics of the respondents.

	Total (n = 2,533)		Host (n = 1,182)		Refugee (n = 1,351)		p value^3^
Number (N), Percentage (%), otherwise stated							
**Women**							
District							
Arua	1,273	50.3	575	48.6	698	51.7	
Kiryandongo	1,260	49.7	607	51.4	653	48.3	ns
Age (in years)	2,533	25	1,182	25	1,351	25	ns
Education level							
None	636	25.1	217	18.4	419	31	
Primary	1,512	59.7	799	67.6	713	52.8	
Secondary and above	385	15.2	166	14	219	16.2	<0.001
Marital status							
Not married or in union	526	20.8	188	15.9	338	25	
Married or in union	2,007	79.2	994	84.1	1,013	75	<0.001
Number of children	2,533	2	1,182	2	1,351	2	ns
Delivered a child in Uganda in the last two years							
No	1,694	66.9	708	59.9	986	73	
Yes	839	33.1	474	40.1	365	27	<0.001
Engaged in paid work during the last year							
Yes	1,260	49.7	338	28.6	922	68.2	
No	1,273	50.3	844	71.4	429	31.8	<0.001
**Household**							
Household size							
< 9	1,853	73.2	956	80.9	897	66.4	
> = 9	680	26.9	226	19.1	454	33.6	<0.001
Household received cash or in-kind benefits from NGO/UN/Government programmes							
No	1,156	45.6	1,156	97.8	0	4	
Yes	1,377	54.4	26	2.2	1,351	100	<0.001
Household food insecurity in last month^1^							
No	1,188	46.9	708	59.9	480	35.5	
Yes	1,345	53.1	474	40.1	871	64.5	<0.001
Gender of household head							
Male	1,280	50.5	920	77.8	360	26.6	
Female	1,253	49.5	262	22.2	991	73.4	<0.001
Household consumption expenditure^2^ (UGX) over the last month	2,533	198,548	1,182	193,309	1,351	203,132	<0.10

^1^Household is categorised as food insecure if any member in the household went a whole day and night without eating anything in the previous month.

^2^Consumption expenditure estimates are the self-reported values to the question "What was your household expenditure during the last month"?

^3^p-values are derived from χ^2^ tests for categorical variables and t-tests for continuous variables

Hosts were more likely to be married (84.1%, p value <0.001) and had a higher number of women who gave birth during the last two years (40.10%) compared to refugees (27%) (Table 4).

### Utilisation and costs of antenatal care

Almost all (97%) women who reported giving birth in the two years prior to the survey had at least one ANC visit during their last pregnancy, and over 70% (compared to 60% from DHS, 2016 (37)) had four or more visits during their pregnancy, with no evidence of a difference between refugee and hosts. Almost all women attended a health centre for seeking ANC (Table 5).

**Table 5:** Utilisation and costs associated with antenatal care for refugees and host populations in Arua and Kiryandongo.

	Total (N = 839)		Host (n = 474)		Refugee (N = 365)		p value^2^
* *Number (N), Percentage (%), otherwise stated							
Had at least one ANC visit in last pregnancy	814	97	456	96.2	358	98.1	ns
* Of which*:							
Had 4 or more ANC visits in the last pregnancy	578	71.01	323	70.83	255	71.23	ns
Accessed ANC during the first trimester	353	43.37	194	42.54	159	44.41	ns
*Of which*:							
ANC location							
Host Health Centre	365	45	361	79.1	4	1.1	
Refugee Health Centre	407	50	55	12	352	98.3	
Hospital	30	3.7	29	6.4	1	0.3	
Private Clinic	5	0.61	4	0.88	1	0.28	
Other^1^	7	0.9	7	1.55	0	0	<0.001
Paid medical fees for ANC	32	3.9	28	6.1	4	1.1	<0.001
Mean payment in (UGX)	32	16,144	28	16,043	4	16,850	ns

^1^Includes informal visits of community health workers, local women, family members, traditional birth attendants etc.

^2^p-values are derived from χ^2^ tests for categorical variables and t-tests for continuous variables

Even though most host women attended ANC at host health centres (79.10%) and refugee women attended at refugee health centres (98.3%), more host women (12%) attended refugee health centres compared to refugee women attending host health centres (1.1%). A higher proportion of host women accessed ANC at a hospital (6.4%) than refugees (0.3%) (Table 5).

These findings are partially explained by the role of incentives. Many respondents noted that nutritional and food supplements provided at each ANC visit at refugee health facilities and the delivery kits (e.g. Mama kits and Dignity kits) are key to incentivising both host and refugee women seeking ANC.

*“What motivates them [pregnant women] are the incentives*. *Like if we have Mama kits and then delivery kits in general*, *they come readily and then food items because also some nutrition programme*. *So*, *they come easily and that attracts more nationals also to come to access ANC services*. *But when these incentives are not there*, *they*, *the uptake is very low”*. (KI 111, NGO, Arua)

Despite high overall ANC coverage, respondents also noted a range of barriers facing both host and refugee women seeking ANC, which included a lack of knowledge of ANC, lack of available female providers (resistance to see male providers), distance to lower-level health facilities in particular for host women living outside of refugee settlements, as well as misinformation and language barriers experienced mostly by refugee populations and by some host populations who do not speak English.

“*So*, *a mother comes to you speaking a language you don’t understand*. *You will have to tell her go look for an interpreter*. *When you tell her to go look for an interpreter maybe she can think you have chased her away*. *Well they [interpreters] were there last year but the funds maybe were not favouring them so they were laid off until now*. *This year is going to end*, *we have struggled with language*. *In fact*, *it also delays our service delivery*.*”* (KI 123, midwife, Kiryandongo)

“*For ANC*, *[refugee and host] women are coming*, *people are coming though they come late… I may say a misconception in the community [is] that maybe when a woman is still in the first trimester because the tummy is not yet big*, *it is appearing like someone who is not pregnant*. *They think that when you come for ANC*, *you will be chased away*. *They [health workers] will tell you that you are not pregnant because there is no evidence your stomach*, *your tummy is not … but that is the challenge we have*, *they come late for ANC*. *Usually from the second trimester*, *that is when they are coming for their first ANC*.*”* (KI 121, clinical officer, Arua)

Refugee and host men also reported the challenge of taking their partners to ANC visits when they are scheduled in the middle of working days.

“*The other challenge is that the days the tell the women to go for antenatal don’t favour some men such as Monday and Thursday*. *These don’t favour the working class because you have also to be on duty yet madam [wife] is pestering you ‘please take me to hospital*.*’ Here you have to always get permission from your boss to be off duty and they are not amused*.*”* (FGD 21, host male, Kiryandongo).

**Fig 2:**
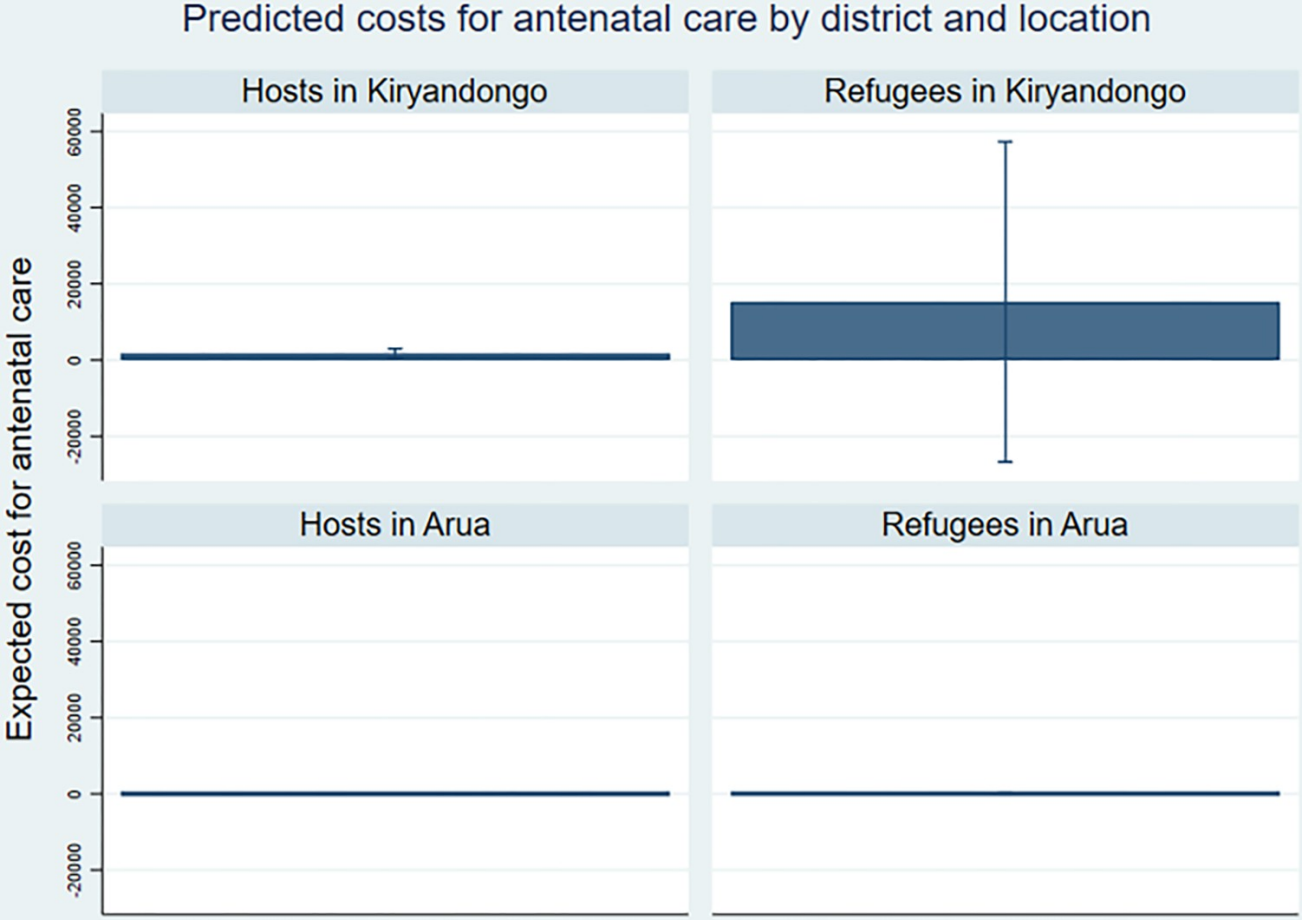
Predicted costs for antenatal care by district and location.

Very few women (4%) paid for ANC (Table 5), and refugees were 5.4% less likely to pay for ANC compared to hosts (Table 9). The costs were UGX 1,000 less for the refugees who paid than the hosts. Male and female respondents explained that ANC at refugee and governmental host health facilities is free of cost, but that pregnant women are asked to pay for gloves and a “*kavera [plastic sheet] for you to lie on as they are examining you*” (FGD 9, host woman, Kiryandongo). Many respondents also cited transport costs required for accessing ANC, though this was not measured in our survey. Fig 2 shows that expected costs of accessing antenatal care was highest among the refugees in Kiryandongo when adjusted for the probability to pay, with the population in Kiryandongo being 3.8% more likely to pay for ANC than those in Arua (Table 9).

### Utilisation and costs of facility-based delivery care

Overall rates of institutional delivery were almost 90% (compared to 70% from DHS, 2016 (37)), with refugees being 7% more likely to deliver in facilities compared to hosts upon controlling for the other characteristics (Table 8, p <0.001). Table 8 also indicates that women with lesser number of children, and therefore lesser experience in delivery are more likely to deliver at a health facility. Several key informants agreed that an increasing number of women want to deliver in health facilities “because of that Mama kit, because of qualified skilled midwives who are now available in the health centres” (KI 136, midwife, Arua), which contrasts with ANC where respondents expressed concern over lack of skilled female staff. Most women gave birth in health centres (90.0%), with a higher proportion of hosts delivering at hospitals (11.0% vs 4.0%) (Table 6). A considerable proportion (13.6%, n = 54) of host women delivered at a refugee health centre, whereas few refugee women delivered in host health centres (1.16%, n = 4) (Table 6). This can be explained by qualitative research findings that refugee health centres are better staffed and equipped compared to host health centres, due to the additional funding they receive from humanitarian donors and NGOs. For example, a health centre in Kiryandongo refugee settlement received external donor funding to equip it to perform caesarean sections, which led to host women “*coming from neighbouring districts for caesarean sections because it is free*… *other Government hospitals like [name anonymised] and where*, *they may charge you money*” (KI 122, health facility manager, Kiryandongo).

**Table 6:** Utilisation and fees associated with facility-based deliveries for refugee and host women who delivered in Arua and Kiryandongo in the 2 years prior to the survey.

	Total (N = 839)		Host (N = 474)		Refugee (N = 365)		p value^1^
Number (N), Percentage (%), otherwise stated							
Facility based delivery for last pregnancy in past 2 years	741	88.3	396	83.5	345	94.5	<0.001
Place of delivery							
Hospital	58	7.83	45	11.36	13	3.77	
Refugee Health Centre	380	51.28	54	13.64	326	94.49	
Host Health centre	293	39.54	289	72.98	4	1.16	
Private Clinic	10	1.35	8	2.02	2	0.58	<0.001
*Of which paid for*:							
Fees	57	7.69	48	12.12	9	2.61	<0.001
Mean payment (UGX)	57	50,807	48	34,188	9	139,444	<0.05
Drugs	8	1.08	6	1.52	2	0.58	ns
Mean payment (UGX)	8	63,788	6	80,050	2	15,000	ns
Delivery Kits	279	37.7	127	32.1	152	44.1	<0.001
Mean payment (UGX)	279	17,033	127	13,224	152	20,216	<0.001
Medical costs (fees + drugs + kits)	315	42.51	161	40.66	154	44.64	ns
Mean payment (UGX)	315	20,440	161	19,353	154	21,577	<0.10
Other (non-medical) costs	250	33.74	136	34.34	114	33.04	ns
Mean payment (UGX)	250	35,284	136	24,173	114	48,539	<0.001
Transport costs for delivering at the facility	423	57.1	254	64.1	169	49	<0.001
Mean payment (UGX)	423	9,749	258	11,368	170	7,665	<0.001
Total costs for delivering at the facility (medical+ non-medical)	519	70.04	292	73.74	227	65.8	<0.05
Mean payment (UGX)	519	20,352	292	20,372	227	20,325	ns

^1^p-values are derived from χ^2^ tests for categorical variables and t-tests for continuous variables

Refugees were less likely to pay for transport to a facility for delivery care than hosts (49% vs 64.%1 respectively). The transport cost for delivery care among those who paid was also lower for refugees than host populations (UGX 7665 vs 11,368) (Table 6). This can be explained by qualitative research findings that hosts pay to travel longer distances to refugee health centres as they are better staffed and equipped compared to governmental health facilities. Equally fuel stock outs in ambulances were also widely reported in interviews, with host women expected to pay for this cost if needing to be referred to a higher-level health facility during childbirth.

Only 7.7% (n = 57) of women who delivered at a facility paid any user fees for delivery. However, a much higher proportion who paid were host women compared to refugees (12.1% vs. 2%, p <0.001). This difference in likelihood of payment goes away when we control for other characteristics (Table 9). Only 1.0% (n = 8) of women reported paying for drugs during the delivery, despite key informants reporting stock outs in both governmental and refugee health centres, with no difference between refugees and hosts. A higher proportion of refugees (44.1%) compared to hosts (32.1%) had to pay for accessing delivery kits, mean payment also being higher for refugees (by UGX 20,216, p<0.001, Table 3). Refugee women were also counselled in their ANC visits and by Village Health Teams (VHTs) in their communities to buy components of dignity kits which could be out of stock in refugee health centres. About a third of women incurred costs for items such as food, with the cost being twice as high for refugees as hosts (UGX 48,539. Vs 24,173). When all costs are considered, refugee and host women were equally likely to pay for delivery with similar average total costs., Higher transport costs among hosts was offset by the higher costs of dignity kits among refugees.

**Fig 3:**
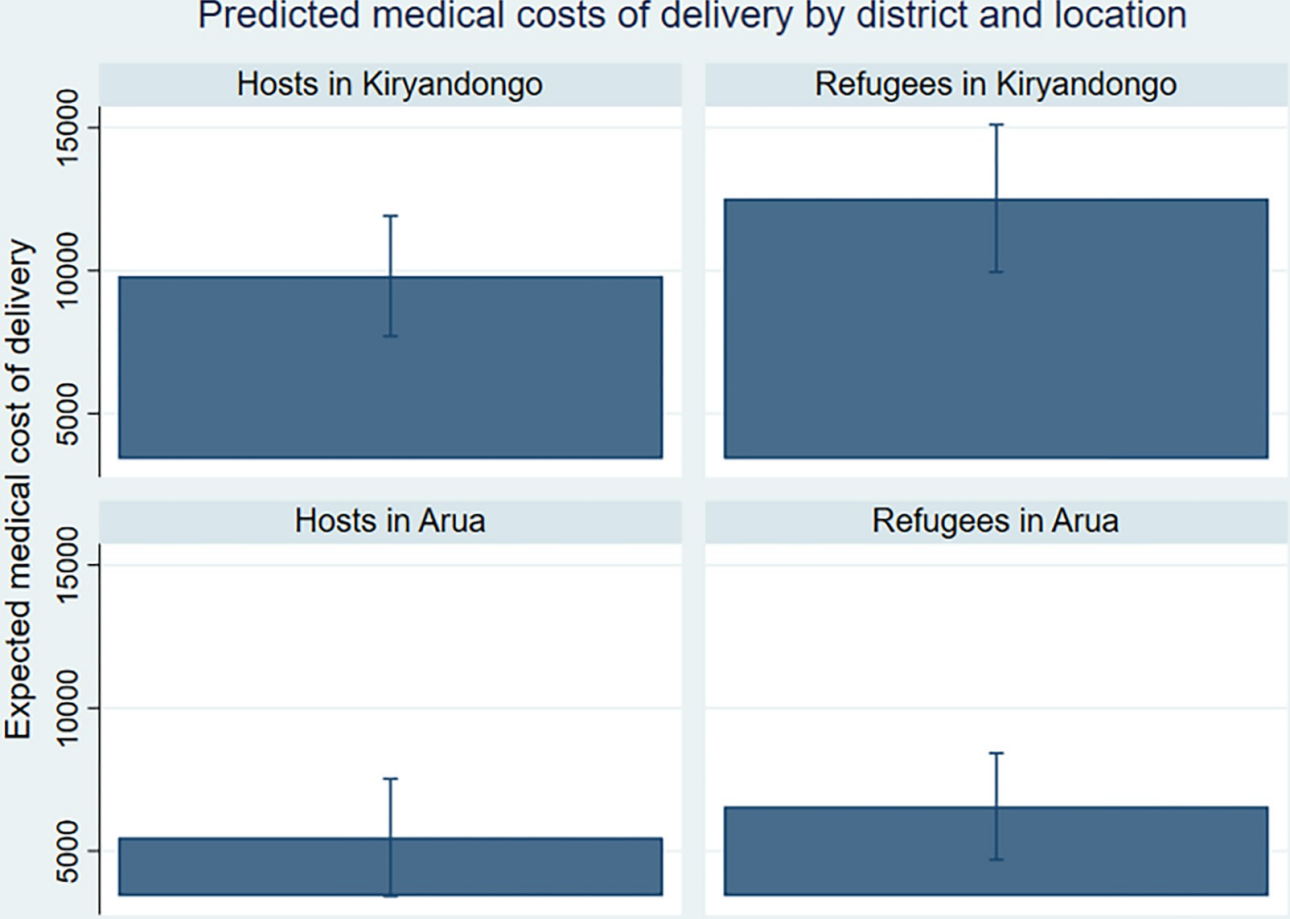
Predicted medical costs of delivery by district and location.

The location of refugee and host communities also affected the costs of delivery care. Although equally likely to incur transport costs, the transport cost of delivery for those who paid was UGX 4000 higher for those living in Arua irrespective of refugee status (Table 9). Qualitative research findings point to distance being an important factor which drove the high transport costs in Arua (more for host communities in Arua than refugees, Fig 3). Although Arua had a higher number of health facilities the communities were further away from the town which housed the Arua Regional Hospital which is a 2.5 hour drive away from the Rhino Camp Refugee Settlement and host communities. Greater distance and time required to reach the referral hospital could influence the transport costs for those who pay in Arua. However, women were 14.3% less likely to pay for medical costs of delivery in Arua compared to those in Kiryandongo, with those who paid paying on average UGX 5000 less (Table 9). Qualitative research findings highlighted there was an influx of humanitarian funding to refugee health centres in Arua because Rhino settlement hosts more recently resettled South Sudanese refugees, whereas Kiryandongo no longer accepts new refugees. A VHT explained, “*if you know your wife has pregnancy*, *you have to buy everything*” (FGD 31, Kiryandongo).

### Utilisation and costs for modern family planning methods

About two thirds (66%, n = 1052) of sexually active and non-pregnant women in the survey were identified to be in need for contraception. Modern family planning usage was more prevalent among host women (95.5%) as compared to refugee women (71.6%, p value <0.01) (Table 7), however, this difference was only marginally significant when controlling for other factors (Table 8). Several key informants noted that “*cultural tradition does not allow them [refugees and Ugandans] to go for family planning*” (KI122, health worker). Most key informants also noted the role of the conflict in South Sudan hindering refugees’ use of modern family planning because “*they have lost very many people [due to conflict]*, *so they do not want to [use it]”* (KI121, health worker).

**Table 7:** Utilisation and fees associated with contraception for host and refugee women in need of contraception in Arua and Kiryandongo.

	Total (N = 1052)		Host (N = 634)		Refugee (N = 418)		p value^1^
Number (N), Percentage (%), otherwise stated							
Number with met need	358	34	224	35.3	134	32.1	ns
*Of which*							
Uses Modern Contraceptive methods	310	86.59	214	95.54	96	71.64	<0.001
*Of which* pays for contraceptives	45	12.6	40	17.9	5	3.7	<0.001
Mean payment (UGX)	45	4,571	40	4,318	5	6,600	ns

p-values are derived from χ^2^ tests for categorical variables and t-tests for continuous variables

**Table 8:** Marginal effects of the multivariate logistic regressions for utilisation outcomes with significant differences between hosts and refugees.

	Delivered at a heath facility	Used modern contraceptives
Refugee population (vs hosts)	0.0720***	-0.0683*
	(0.0244)	(0.0387)
Arua district (vs Kiryandongo)	-0.00565	0.0711**
	(0.0279)	(0.0300)
Age (in years)	0.00424	-0.00453
	(0.00258)	(0.00312)
Primary education (vs secondary education)	-0.0240	-0.103***
	(0.0255)	(0.0395)
No education (vs secondary education)	-0.0395	-0.265***
	(0.0336)	(0.0491)
Married or in union	-0.0211	-0.0836
	(0.0706)	(0.136)
Number of children	-0.0172**	0.0213**
	(0.00770)	(0.0104)
Female household head	0.0375	0.00846
	(0.0246)	(0.0419)
Engaged in a paid activity over the last year	-0.0395	0.0580*
	(0.0283)	(0.0350)
> 9 members in the household	0.0529*	-0.0642**
	(0.0276)	(0.0293)
Household consumption expenditure during the last month (UGX)	-9.16e-08	2.05e-07**
	(6.07e-08)	(8.42e-08)
Observations	828	1,037

Standard errors in parentheses.

*** p<0.01

** p<0.05

* p<0.1

**Fig 4:**
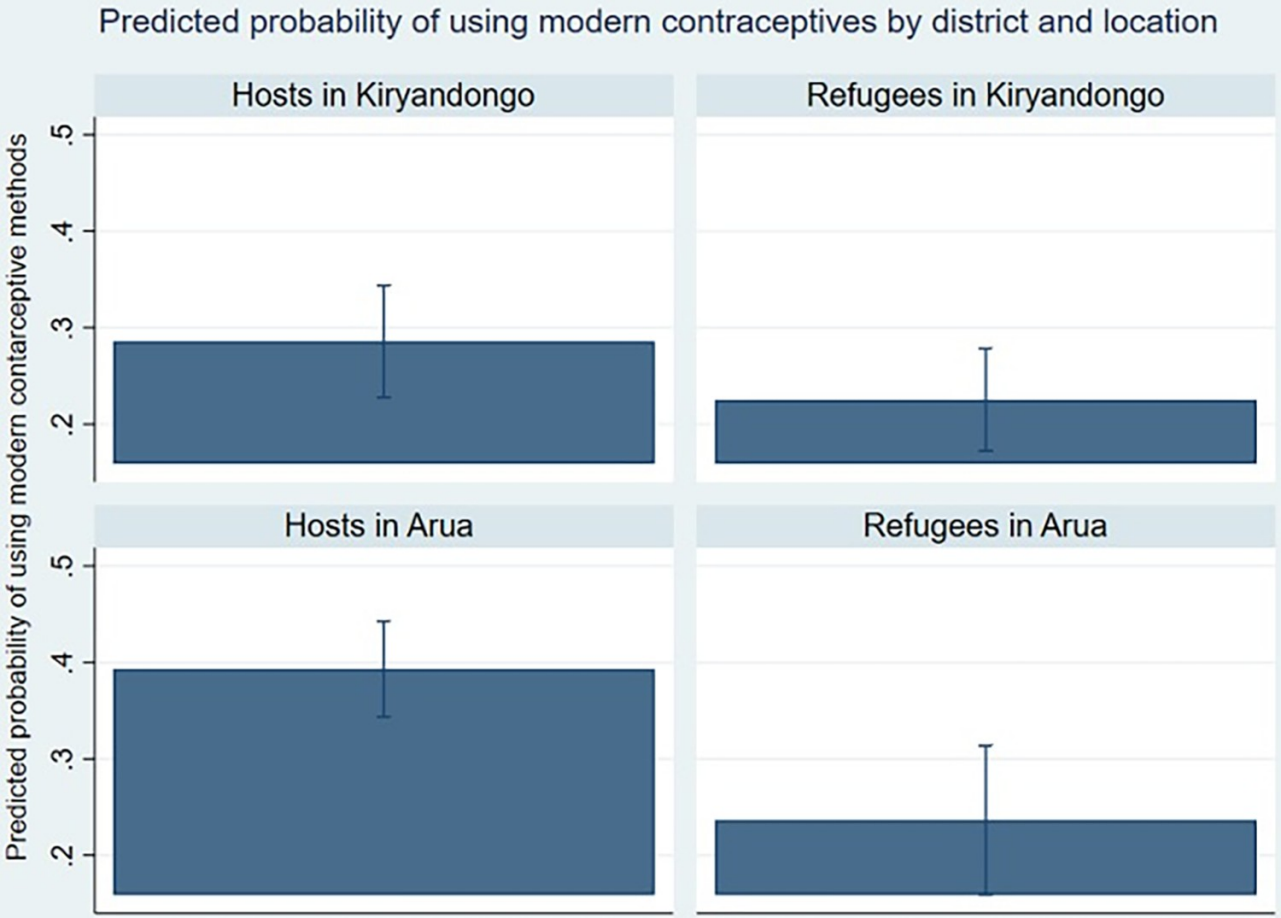
Predicted probability for modern contraceptive use by district and location.

In Table 8, our results show that education and household size play a more significant role in determining use of family planning methods, as the effect on refugee status is only borderline significant. Respondents in Arua were 7% more likely to use modern contraceptives (Table 8) (particularly the host women, Fig 4) compared to Kiryandongo.

Key informants reported barriers to modern family planning use relating to access, quality of services, health concerns and family/community opposition, all which emphasize the importance of men’s gendered roles in relationships, cultural and religious beliefs and lack of agency for most women to make their own decisions about reproductive health and choices.

**Fig 5:**
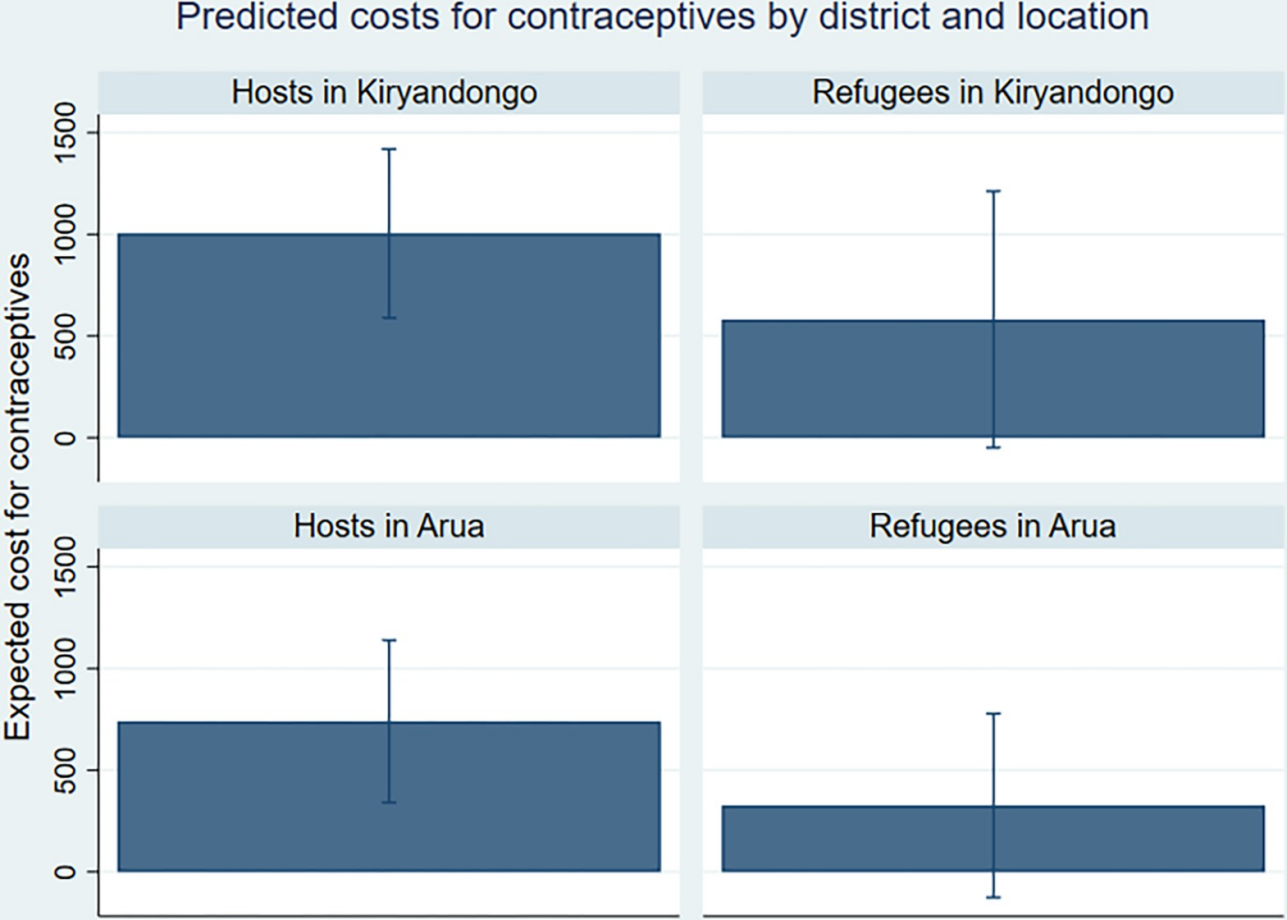
Predicted costs for modern contraceptive methods by district and location.

Very few (12.5%, n = 45) women paid for contraceptives, which can be explained by contraception being free in governmental and refugee health centres. However, refugees were 17.6% less likely to pay than hosts (Table 9). Expected costs of contraceptive methods was higher for refugee women in Kiryandongo than in Arua (Fig 5). Host women in Kiryandongo were likely to pay the highest price for contraceptive methods. Both refugees and host populations widely agreed that factors affecting their access to modern family planning include distance to the health facility, in addition to the long queues from “*morning to nine hours or evening” and lack of privacy that they encountered at governmental health facility that made going to private clinics more appealing* (KI2, female refugee).

**Table 9:** Two-part model results for the cost of antenatal care, delivery care and contraceptives.

	**Antenatal costs**		**Medical costs for delivery**		**Transport cost for delivery**		**Total delivery costs**		**Costs of contraceptives**	
	Probability to pay	Costs^1^	Probability to pay	Costs	Probability to pay	Costs	Probability to pay	Costs	Probability to pay	Costs
Refugee population (vs hosts)	-0.0541***	-1,009*	0.0615	3,111**	-0.143***	-3,584***	-0.0629	-548.2	-0.176***	-519.4
	(0.0200)	(575.7)	(0.0516)	(1,348)	(0.0541)	(934.7)	(0.0487)	(1,744)	(0.0534)	(406.4)
Arua district (vs Kiryandongo)	-0.0377**	-840.7**	-0.143***	-4,844***	-0.0539	4,017***	-0.0796*	-610.4	-0.0263	128.5
	(0.0156)	(387.6)	(0.0403)	(1,062)	(0.0530)	(879.6)	(0.0424)	(1,444)	(0.0449)	(351.4)
Age (in years)	0.000799	-37.24	-0.00151	-75.34	-0.00983**	-38.96	-0.00919**	-43.93	0.00813**	83.01**
	(0.00138)	(61.00)	(0.00447)	(130.2)	(0.00477)	(87.74)	(0.00432)	(191.3)	(0.00388)	(36.79)
Primary education (vs secondary education)	-0.0239	-858.1	0.0262	-778.0	0.00734	-32.20	0.0229	-1,426	-0.0469	-555.3
	(0.0276)	(814.5)	(0.0562)	(1,713)	(0.0657)	(1,079)	(0.0516)	(2,177)	(0.0556)	(402.8)
No education (vs secondary education)	-0.0310	-999.5	0.0829	14.07	0.0356	1,486	0.0320	541.6		
	(0.0302)	(908.7)	(0.0650)	(1,910)	(0.0702)	(1,218)	(0.0605)	(2,786)		
Married or in union	-0.0421	-839.1	-0.0616	-546.0	0.0490	775.2	0.0209	2,132		
	(0.0527)	(1,328)	(0.120)	(2,875)	(0.125)	(2,063)	(0.123)	(3,173)		
Number of children	0.000489	197.0	0.0108	48.65	0.0173	-116.8	0.0212	-151.4	0.00324	-57.81
	(0.00629)	(258.2)	(0.0156)	(427.2)	(0.0172)	(289.0)	(0.0173)	(643.3)	(0.0156)	(102.7)
Female household head	0.00927	294.2	-0.0453	-2,249**	-0.0379	-739.5	-0.0532	-2,867*	0.0419	269.7
	(0.0181)	(715.1)	(0.0453)	(1,101)	(0.0423)	(651.2)	(0.0393)	(1,586)	(0.0783)	(418.9)
Engaged in a paid activity over the last year	-0.00791	-208.6	-0.0114	-1,650	0.00951	324.0	-0.00868	-1,006	-0.0752*	-650.7**
	(0.0191)	(376.5)	(0.0420)	(1,294)	(0.0423)	(722.9)	(0.0374)	(1,680)	(0.0449)	(319.6)
> 9 members in the household	-0.00369	-269.0	-0.0284	-2,097*	0.0352	887.2	0.0267	-1,401	0.0338	-72.43
	(0.0161)	(384.8)	(0.0403)	(1,098)	(0.0417)	(783.9)	(0.0394)	(1,481)	(0.0464)	(237.3)
Household consumption expenditure during the last month (UGX)	8.31e-09	0.00044	-5.08e-08	0.00141	1.18e-07	0.000272	1.34e-08	0.00288	-9.03e-08	-0.000320
	(3.66e-08)	(0.000937)	(1.12e-07)	(0.00350)	(1.39e-07)	(0.00215)	(1.26e-07)	(0.00418)	(1.06e-07)	(0.000833)
Observations	805	805	732	732	732	732	732	732	293	293

Table outlines the results of Two-Part model including the probability to pay (logit estimates) and the costs for those who pay (GLM) for antenatal and delivery care. Standard errors have been clustered at the village level.

*** p<0.01

** p<0.05

* p<0.1

^1^The costs estimates are adjusted for the probability of paying for the service

“*Because aah if you ask them*, *they say aah we prefer going to the private clinic*

*The queue is not there”*. (KI114, NGO, Arua)

### Characteristics influencing differences in healthcare utilisation outcomes

Tables 8 and 9 present results on various characteristics influencing differences in healthcare utilisation outcomes amongst refugees and host populations in both study settings.

## Discussion

Our study found that overall coverage of SRMH services was higher compared to the national average for both host and refugee women in Arua and Kiryandongo districts of Northern Uganda. Similar to three previous studies set in Uganda [5, 33, 34], we find that refugee women are more likely to deliver at a health facility compared to host populations. Higher rates of institutional delivery among refugees can be explained by refugee health centres generally being well equipped and staffed–often with generous financial support from humanitarian actors to supplement government funding, offering incentives to patients, including Mama kits and Dignity kits, and used by both populations. However, in contrast to previous literature which reported lower refugee access to ANC in Uganda, we found that ANC access was high and similar for refugees and hosts in Arua and Kiryandongo, potentially due to strong engagement and social mobilisation of refugee communities by the Government and other development partners over recent years, as well as the incentive of a nutritional supplement when women attend ANC. Our estimates are slightly higher than those reported by King et al, 2022 as we use data from pregnancies over the last 2 years as opposed to the last year pregnancies [7]. The increased resourcing of refugee health centres in terms of staff, drugs and laboratory equipment, relative to that of hosts was highlighted in previous research [8]–suggesting that the discrepancy in resourcing of refugee compared to host health centres has persisted for more than 10 years. Differential treatment of refugees and discrimination has been reported elsewhere [5] but was not reported to be an issue in our study, with the main access barrier being that of language and an absence of female providers for ANC.

Ours is one of the few studies to assess the cost burden of SRMH care among refugees and host populations in low- and middle-income countries [35]. We found that refugees were less likely to pay for ANC and family planning, but they were more likely to pay for deliveries in terms of delivery kits and food, though transport costs for deliveries were lower. Our results support the benefits of delivery kits for financial protection and institutional delivery. Qualitative findings indicate that kits served as an incentive for refugees to deliver at a facility. When the kits were out of stock due to high demand, patients paid for them out of pocket, which was an issue for refugee health centres reliant on donor funded Dignity kits. This suggests that care delivered to refugees is not consistently better and heterogeneity in quality of care exists even within refugee health centres. A previous World Bank study [11] also found that refugees pay less for health care due to the presence of health NGOs and humanitarian organisations in refugee settlements. Host communities generally faced higher transport costs than refugees due to ambulance shortages and longer distances to reach health facilities.

Even though the use of family planning services remained low for both host and refugee women, refugee women were less likely to use and buy modern contraceptives as compared to host women in our study. These findings are consistent with Bakesiima et al. 2020 [36]. Our study’s quantitative findings identify education and household size playing a more significant role in determining use of family planning methods for both populations, with the effect of refugee status found to be only marginally significant. Main reasons for both refugees and host populations not using modern contraceptive methods identified in the qualitative data included perceived dangers to health and family/community opposition, cultural and religious beliefs, and lack of agency for most women to make their own decisions about their reproductive health and choices [37]. Some refugee women also reported being more reluctant to use and pay for contraception because of individual, family and community pressures to “replace” lives lost in war, as well as social norms restricting the use of contraceptives.

We also found significant differences in costs of seeking care by geographical location. Greater distance and time required to reach the referral health facilities influenced the cost of seeking care in Arua whilst lower quality of services and stock outs at health centres in Kiryandongo increased medical costs, especially for host women. This highlights how heterogeneity of context affects access for refugee and host communities within the same country, and the advantages proffered to both communities benefiting from donor investments in health facilities.

The issue of differential access to SRMH care by host communities and imbalances in health services available to refugees compared to hosts needs careful consideration when designing the health systems response to refugees. Under CRRF, 30% of donor funding for refugee advancement and settlement response should be spent for the development of host communities. However, this funding is scarce and growing refugee and host population numbers put significant pressure on the health system serving both populations. Further, funding is uneven for refugee health centres and varies considerably by location, type of service provided and is influenced by donor objectives rather than local priorities. The decreasing trend of government healthcare spending as a share of total Gross Domestic Product [13] is likely to negatively impact both hosts and refugees’ access to care [36], and potentially further widen the gap between hosts and refugees, in areas where refugee facilities continue to benefit from additional humanitarian funding.

Our findings suggest that additional investment in health centres located in refugee communities have offered refugees an advantage in terms of access to delivery care, signalling inequitable access to health care services between refugee and host populations in the study settings. Greater health systems investment in facilities in host areas in terms of infrastructure, supplies and human resources would help to close this gap by further increasing access and affordability of health care in host communities [38, 39].Delivery kits comprise a substantial share of the costs of delivery incurred by women, and providing these for free makes this service more affordable. However, transport and food costs should not be overlooked, and financing mechanisms to cover non-medical costs are also needed to enhance progress to UHC [40]. Also, supply-side investments will need to be complemented by demand-side strategies to increase uptake of modern contraceptives, especially among refugee populations.

Our study has several strengths. Our findings have been informed by rigorous evidence, collected in the form of a well powered quantitative survey, FGDs and SSIs. Ours is also the first mixed method study, evaluating the level of utilisation and affordability of key SRMH services across two unique geographical locations, for both refugee and host women in Northern Uganda.

However, our results should also be interpreted with caution for several reasons. First, the costs reported in the study have been self-reported by the women and in one third of cases, by adolescent girls. Even though we minimized recall bias in the survey by following the standard practice of one-month recall period, they may not always be accurate, especially in case of adolescents who may not have a complete overview of household expenses. Second, random sampling ensures that our findings are representative of the districts under question, but they may not be generalizable to other regions of Uganda where geography, composition of host and refugee population and availability of services are considerably different as compared to Arua and Kiryandongo. Also, these findings are limited to rural settings are not generalizable to urban contexts. Third, the data collection happened in July 2019, which was before the COVID-19 pandemic. We do not have evidence on how the affordability and utilisation of SRMH services might have changed following the pandemic and encourage future research to evaluate this [41]. Fourth, we did not identify the type of delivery, and some women may have had a c-section which would influence costs incurred, but we were unable to control for this in our analysis. Further, our qualitative data suggest that transport costs associated with ANC and family planning services would have contributed to the overall cost and may have differed across refugee and host populations.

## Conclusions

We found high levels of access to maternal care services among refugee and host communities in Northern Uganda, but with lower levels of met need for family planning. Refugees had higher delivery care access than host communities. They also incurred higher costs for delivery kits and food but less for transport. Higher relative investments in refugee health centres contribute to better access to delivery care in both study settings. Greater investment to increase the number of host facilities and the quality of service provision in the form of infrastructure, supplies and human resources is needed to further enhance maternal care access among host communities, together with ongoing funding of delivery kits across all communities, and financing mechanisms to support non-medical costs for deliveries which can be substantial. Efforts to meet both refugee and host communities’ desired family planning needs are urgently needed, ideally by co-designing tailored strategies with women, their partners and communities to take into account varying religious, social and cultural beliefs and contexts.
